# CD146, from a melanoma cell adhesion molecule to a signaling receptor

**DOI:** 10.1038/s41392-020-00259-8

**Published:** 2020-08-11

**Authors:** Zhaoqing Wang, Qingji Xu, Nengwei Zhang, Xuemei Du, Guangzhong Xu, Xiyun Yan

**Affiliations:** 1 Key Laboratory of Protein and Peptide Pharmaceuticals, Institute of Biophysics, Chinese Academy of Sciences, 100101 Beijing, China; 2 College of Life Science, University of Chinese Academy of Sciences, 100049 Beijing, China; 3 Department of Gastrointestinal Hepatobiliary Tumor Surgery, Beijing Shijitan Hospital, Capital Medical University, 100038 Beijing, China; 4 Departments of Pathology, Beijing Shijitan Hospital, Capital Medical University, 100038 Beijing, China; 5 Nanozyme Medical Center, School of Basic Medical Sciences, Zhengzhou University, Zhengzhou, 450001 China

**Keywords:** Cell biology, Tumour angiogenesis

## Abstract

CD146 was originally identified as a melanoma cell adhesion molecule (MCAM) and highly expressed in many tumors and endothelial cells. However, the evidence that CD146 acts as an adhesion molecule to mediate a homophilic adhesion through the direct interactions between CD146 and itself is still lacking. Recent evidence revealed that CD146 is not merely an adhesion molecule, but also a cellular surface receptor of miscellaneous ligands, including some growth factors and extracellular matrixes. Through the bidirectional interactions with its ligands, CD146 is actively involved in numerous physiological and pathological processes of cells. Overexpression of CD146 can be observed in most of malignancies and is implicated in nearly every step of the development and progression of cancers, especially vascular and lymphatic metastasis. Thus, immunotherapy against CD146 would provide a promising strategy to inhibit metastasis, which accounts for the majority of cancer-associated deaths. Therefore, to deepen the understanding of CD146, we review the reports describing the newly identified ligands of CD146 and discuss the implications of these findings in establishing novel strategies for cancer therapy.

## Introduction

In 1987, Johnson et al. first found that a tumor antigen, MUC18, was expressed most strongly on metastatic lesions and advanced primary melanoma with rare detection in benign lesions. Due to the high sequence homology between MUC18 with cell adhesion molecules (CAMs), this melanoma antigen was given an official name, melanoma CAM (MCAM).^1^ With an increasing number of discoveries about MCAM by various research groups, more alias names were given to this protein, including P1H12, MUC18, A32 antigen, S-Endo-1, Mel-CAM, MET-CAM, HEMCAM, or CD146.^1–3^

CAM is a kind of proteins located on the cell surface and mediates contacting and binding of cell to cell or cell to extracellular matrix (ECM).^4^ These dynamic interactions provide signals input into the cellular decision-making process such as cell growth, survival, migration, and differentiation,^5^ essential for embryonic development and for maintaining the integrity of tissue architecture in adults.^6,7^ Dependent on adhesion, some CAMs can initiate the formation of complexes composed of extracellular ligands, kinases, and cytoskeletal proteins.^8^ Abnormal expression of CAMs can cause various diseases, such as cancer and inflammatory disorders.^9,10^

There are three forms of CD146 proteins in human, mouse, and chicken. The two membrane-anchored forms of CD146 are encoded by *cd146* gene and soluble form of CD146 (sCD146) is generated by the proteolytic cleavage of the membrane forms.^11–13^ Soluble CD146 can be detected in cell culture supernatants, serum, and interstitial fluids from either healthy or unhealthy subjects.^14–16^ Because sCD146 does not have either transmembrane or cytoplasmic regions, it is not competent in cellular adhesion.^17,18^ Therefore, we will not describe sCD146, its ligands and its functions in this review, although it is a potential target in tumor microenvironment of CD146-positive invasive tumors.^19^

Recent evidence has revealed that membrane-bound CD146 may act as a cell-surface receptor to bind with various ligands involved in cellular signaling transduction independent of the adhesion properties. In order to deepen the understanding of the functions of CD146 in physiological and pathological processes, we summarize the various newly identified ligands of CD146 and the ligand-elicited roles in signal transduction and discuss the implications of CD146 in remodeling interactions between the cancerous cells with the elements of their surrounding microenvironments.

## The CD146 protein

Membrane CD146 protein has two isoforms: long form (CD146-l) has a long cytoplasmic tail; short form (CD146-s) has a short cytoplasmic tail.^17,18^ These two CD146 isoforms are produced from different exon splicing strategies and the premature molecules have a signal peptide located on the anterior region of the amino terminal.^20^ In human, mature CD146 protein is composed of an extracellular sections with five distinct Ig-like domains that exist in a V–V–C2–C2–C2 structural motif, a hydrophobic transmembrane region and a short cytoplasmic tail.^21^ The cytoplasmic domain in both isoforms contains two potential recognition sites for protein kinases C (PKC), an ERM (protein complex of ezrin, radixin, and moesin) binding site, a motif with microvilli extension, and a double leucine motif for baso-lateral targeting.^21^ The two isoforms co-exist as monomers and dimers and the dimerization is mediated through a disulfide bond between cysteine residues in the C2 domain most proximal to the membrane.^20,22^ However, the information about CD146 crystal structure, including secondary and tertiary, is still lacking.

CD146 is a highly glycosylated type I transmembrane protein and belongs to the immunoglobulin superfamily. Based on bioinformation analysis, eight putative *N*-glycosylation sites are present in the extracellular fragment across species.^23^ In clear cell renal cell carcinoma and prostate cancer, CD146 glycosylation levels were upregulated.^24,25^ In 2018, it was reported that CD146 glycosylation is favorably carried out by b-1,3-galactosyl-Oglycosyl-glycoprotein b-1,6-*N*-acetylglucosaminyltransferase-3, which was overexpressed in highly metastatic melanomas. Such glycosylations can extend CD146 protein stability, upregulate CD146 protein levels, and lead to elevation of CD146-mediated cellular motility in melanoma cells.^26^ These observations suggest that the degree of CD146 glycosylation may be directly related to malignant progression of tumors, especially CD146-positive neoplasms.

## The expression profile of CD146 protein

Based on literature, metazoan CD146 has been detected in majority of cell types, including vessel constituting cells (endothelium, pericyte, and smooth muscle cell), epithelia, fibroblasts, mesenchymal stem cells, and lymphocytes, except erythrocytes.^21^ Under physiological conditions, CD146 expression is restricted to limited adult normal tissues and its adhesive strength is relatively weak, in contrast to most other CAMs, which show wide expression patterns in normal adult tissues and strong adhesion strength.^21,23^ However, CD146 expression is broadly and highly detected in embryonic tissues compared to its abundance in normal adult tissues.^21^ In quickly proliferating cells, increased expression of CD146 may allow cells to actively interact with each other and with the elements of the cellular microenvironment, promoting cell proliferation, and migration.

Under pathological conditions, such as inflammation and tumorigenesis, CD146 was upregulated in the related cells and has been identified as a reliable marker for numerous types of cancers. Accumulating evidence shows that CD146 overexpression has been linked to either the initial development of the primary lesion or progression to metastases of most of cancer types, primarily including melanoma,^1,27–29^ breast,^6,30,31^ ovarian,^32–35^ lung,^36,37^ prostate,^38–40^ glioma,^41^ kidney,^42^ hepatic,^43,44^ and gastric cancers.^21,45^ In 2017, Nollet et al. reported that TsCD146 mAb (for tumor specific anti-CD146 monoclonal antibody) can specifically recognize CD146 expressed in cancer cells but not CD146 in physiological vessels, suggesting that structural features of cancer CD146 differ from those of physiological CD146.^28^

## Recognition of CD146 ligands in history

The recognition of CD146 ligands and analysis of their functions was undertaken over a prolonged period in history. Because CD146 is highly expressed in vessel cells and cancer cells, it is likely that CD146 within these cells contributes to cancer metastasis through the mediation of a homophilic adhesion between cancerous cells and vascular endothelia, a key part of the metastatic process. However, evidence of the direct interactions between CD146 and itself is still lacking.^46–48^ Accordingly, it is possible that CD146-mediated adhesion between cancerous cells with vascular endothelia as well as with their surrounding elements occurs through the bidirectional heterophilic interactions between CD146 with its ligands, but not the homophilic interaction with itself.

In 1991, the first CD146’s ligand was found using chicken smooth muscle cells. Taniura et al. discovered that neurite outgrowth factor (NOF) was a ligand of chicken CD146 (Gicerin) and that binding of NOF to CD146 is essential for the development of the chicken retina.^49,50^ However, at that time, due to technological limitations, the molecular characteristics of NOF were not determined. In 2012, Laminin 411 was revealed as the ligand of CD146, facilitating the entry of blood lymphocytes into the central nervous system (CNS). In this report, the authors claimed that Laminin 411 is a major tissue ligand for CD146 on lymphocytes.^51^ In 2014, Ishikawa et al. finally determined the identity of NOF, Laminin 421, which has the same α4 subunit as Laminin 411.^52^

In 2012, our laboratory identified that CD146 can bind with vascular endothelial growth factor receptor 2 (VEGFR2) as a co-receptor required for the activation by vascular endothelial growth factor-A (VEGF-A).^53^ Because VEGF-A is a well-known growth factor with strong pro-angiogenesis effects, this finding provided the mechanism underlying the roles of CD146 in tumor angiogenesis, especially in sprouting stage. Subsequently, our laboratory identified an array of pro-angiogenetic growth factors, including Wingless/integrase (Wnt)5a,^54^ Netrin-1,^55^ fibroblast growth factor (FGF)4,^56^ VEGF-C,^57^ and Wnt1,^58^ as the ligands of CD146. In 2017, we further identified that CD146 on endothelia can directly bind with platelet-derived growth factor receptor-β (PDGFR-β) on pericyte, required for PDGF-B-induced PDGFR-β activation.^59^ Because PDGF-B/PDGFR-β plays crucial roles in recruiting adjacent pericytes to the endothelia, this finding indicates that CD146 is required for vessel integrity.

Until now, there had been a total of 13 molecules or complexes identified as the CD146 ligands (Table 1). According to the characteristics of these ligands, they can be categorized into three groups: components of the ECM, pro-angiogenic factor receptors, and growth factors. All these ligands have been sown to directly interact with CD146 in physiological and pathological processes are involved in the promotion of CD146-mediated angiogenesis and tumor metastasis. Here, we will review the various CD146′ heterophilic ligands and discuss the implications of these findings in tumoral context.

**Table 1:** CD146 ligands

Ligands	Function	Time of discovery	References
Laminin 411	Facilitates lymphocytes entry into CNS	2012	^51^
Laminin 421	Improves cancer metastasis via vascular and/or lymphatic vessels	2014	^52^
Galectin-1	Inhibits cell apoptosis	2013	^107^
Galectin-3	Enhances cell migration and secretion of pro-metastasis cytokines	2017	^122^
S100A8/A9	Helps lung tropic metastasis	2016	^175^
Matriptase	Promotes neuron differentiation	2017	^189^
VEGFR2	Pro-angiogenesis	2012	^53^
PDGFR-β	Control of vascular vessel integrity	2017	^59^
Wnt5a	Enables cell migration	2013	^54^
Wnt1	Promotes fibroblast activation	2018	^58^
Netrin-1	Pro-angiogenesis	2015	^55^
FGF4	Promotes cell polarity establishment	2017	^56^
VEGF-C	Mediates sprouting during lymphangiogenesis	2017	^57^

## CD146 is the receptor of proteins in relation to the ECM

**Fig. 1:**
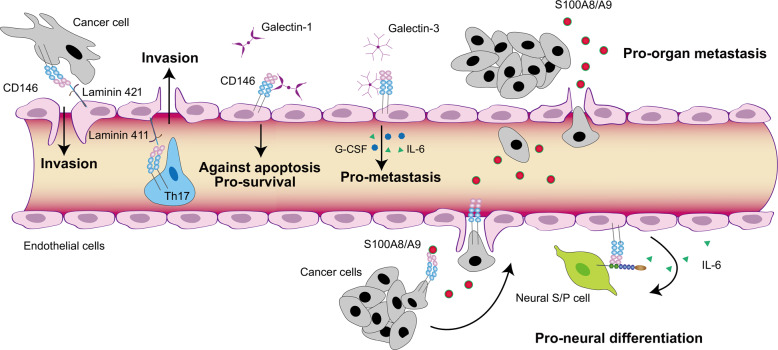
Schematic representation of CD146 as the receptor of proteins related in the ECM. CD146-ligand binding-induced events are indicated by the red arrows. The immunoglobulin domains of CD146 are illustrated as balls and the pink and blue color represent the V and C2 immunoglobulin domains, respectively. The green rectangle and the yellow trapezoid represent the PKC phosphorylation site and the dileucine motif, respectively

One of the critical features of malignant proliferation is cancer metastatic plasticity affected by its microenvironment. This plasticity is a major reason for the failure of inhibition of cancer metastasis. The metastatic process involves epithelial mesenchymal transition (EMT), attachment of metastatic cells to the endothelium of the vascular or lymphatic vessels, and invasion into distant metastatic tissues.^60^ It is well known that the aberrant high expression of CD146 is involved in nearly every step of development and progression in almost all types of malignant cancers.^21^ The findings that several ECM-related proteins, including Laminin 411 and 421, Galectin-1 and -3, S100A8/A9, and matriptase, are specific ligands of CD146, may elucidate the mechanism underlying the function of CD146 in remodeling tumor microenvironments during tumor development, especially metastasis via vascular and lymphatic vessels (Fig. 1).

### Laminins 411 and 421

Laminins are a family of large heterotrimeric αβγ proteins with over 15 different isoforms. Five laminin α chains (α1–α5), four laminin β chains (β1–β4), and three laminin γ chains (γ1–γ3) constitute αβγ heterotrimers. They are denominated according to their chain composition; for example, laminin α4β1γ1 is designated as Laminin 411.^61^ Laminins are predominantly found in basement membranes that compartmentalize different tissues and surround blood vessels, nerves, and adipocytes.^62,63^ They play a crucial role in physiological and pathological remodeling of the ECM during angiogenesis, wound healing, embryogenesis, and tumor metastasis. Remodeling of the ECM during metastasis allows tumor cells to invade their surrounding ECM, spread via the vascular or lymphatic circulation, and extravasate into distant organs.

Laminin isoforms, particularly the laminin α chain, are expressed in a cell and tissue specific manner and are distinctly bound by almost ten different integrins and other cell-surface receptors.^62,63^ The α4-laminins are mesenchymal laminins expressed by the cells of mesenchymal origin, such as vascular and lymphatic endothelial cells, pericytes, and leukocytes, and are required for normal development of the cardiovascular and neurological system in mice.^64–66^ Under pathological conditions, α4-laminins are expressed and secreted by various tumor cells, such as melanoma and glioma,^67–72^ or tumor stroma, lymphatic and vascular vessels, nervous system.^69,73,74^

### Laminin 411

Laminin 411 is expressed along the vascular endothelium.^68,73,75^ This laminin isoform is recognized by various integrins, including α6β1, α3β1, α6β4, and αVβ3, which promote the migration of several cell types along vascular or nervous system tracks.^76–80^

In 2012, Laminin 411 on the vascular endothelia was discovered as a specific ligand for CD146 on a subset of human CD4^+^ T helper (Th) cells.^51^ This subset of human T cells expresses CD146 and can enter tissues to promote pathogenic autoimmune responses. To determine the CAMs involved in the migratory capacity of Th17 cells into tissues, researchers used purified Laminin 411 to identify its receptor. In this study, the authors demonstrated that purified CD146-Fc binds to Laminin 411 with high affinity (27 nM) and that this binding disappeared when the endogenous Laminin 411 was specifically deleted. Correspondingly, blocking this binding by CD146 antibody in vivo also reduced Th17 lymphocyte infiltration into the CNS. Therefore, the authors concluded that Laminin 411 is a major tissue ligand for CD146^+^ lymphocyte.

However, the role of Th17 cells in the pathogenesis of malignant tumors is still remains controversial. Some studies revealed that increased percentage of Th17 lymphocytes among cells infiltrating ovarian cancer cells stimulate tumor progression;^81^ whereas other studies showed that Th17 lymphocytes have anticancer activity and can reduce tumor growth and metastasis.^82^ Therefore, the roles of CD146^+^ Th17 cells in cancer development may be worthy of further investigations.

### Laminin 421

CD146 is a reliable biomarker of endothelia and is concentrated at the intercellular junctions of endothelial cells of vessel system.^21^ Most cancer cells, including melanoma, migrate along the abluminal sides of vascular and/or lymphatic vessels, as they disseminate throughout the body.^83^ Laminin 421 is major laminins of along the tumor-dissemination tracks (blood and lymphatic vessels, nerves, and tumor stroma).^84–86^

To determine the mechanism of CD146 roles in metastasis, researchers used melanoma cells to test what laminin isoforms, other than Laminin 411, can bind with the melanoma marker of CD146. Therefore, they used all laminin α chains to examine the binding affinity with human CD146 in a solid-phase ligand binding assay.^87^ Finally, they found that only Laminin 421, of several laminin isoforms, readily bound to CD146, suggesting that Laminin 421 is a primary ligand for CD146 in melanoma. Accordingly, a function-blocking mAb to CD146 inhibited tumor cell migration on Laminin 421, but not on laminins 411 or 521. In addition, this investigation determined that the identity of NOF, previously identified as a ligand for chicken CD146 (gicerin), is actually Laminin 421.

In this study, the authors also determined that Laminin 411 and especially Laminin 421 are capable of stimulating migration of a broad panel of cancer cell lines through a filter. This investigation is consistent with the observation that the α4-laminins, including Laminins 411 and 421, expressed and secreted by various carcinoma cells, have already emerged as “onco-laminins.”^67–72^ Melanoma CD146 binds with Laminin 421 but not 411, whereas lymphocyte CD146 only binds with Laminin 411; suggesting that the epitopes of CD146 on somatic cancer cells are different from those of CD146 on blood lymphocytes. Therefore, the infiltration of CD146^+^ invasive cancers into tumor-dissemination tracks is likely dependent on the interaction between CD146 and Laminin 421, and blocking their binding may affect the efficacy of cell–cell interactions and interfere metastasis.

### Galectin-1 and -3

CD146 is a highly glycosylated junctional CAM involved in the control of vascular vessel integrity. Sequence analysis predicts the presence of eight putative *N*-glycosylation sites at residue positions 56, 418, 449, 467, 508, 518, 527, and 544.^21^ It has been estimated that 35% of the CD146 molecular mass is attributed to glycans.^88^ The galactose residues in glycans can bind with galectins, and such binding can be inhibited by lactose.

Galectins are a family of soluble carbohydrate-binding lectins that modulate cell-to-cell and cell-to-ECM adhesions.^89^ Up to now, 15 galectins have been identified in mammals and 11 are found in humans. Among them, Galectin-1, -3, and -9 are three best-investigated galectins and Galectin-1 and -3 promote tumor development, progression, and immune escape.^90^ Galectin-1 and -3 can hamper antitumor responses and are considered multifunctional targets for cancer therapy.^91,92^ The underlying mechanisms include interfering with drug efficacy/delivery or reducing the antitumor effect of immune cells. For instance, Galectin-1 confers drug resistance via inducing the expression of multidrug resistance protein 1, which in turn helps tumor cells to pump out cytotoxic drugs, facilitating cancer cells to combat anticancer drugs.^93^ Regarding the immunosuppressive effects of Galectin-1 and -3 on T cells, in a mouse melanoma model, targeted inhibition of Galectin-1 enhanced T cell-mediated tumor clearance;^94^ Galectin-3 can neutralize glycosylated IFN-γ in tumor matrices, ablating the immune response to tumors.^95^ To increase overall responsiveness of tumors to chemo- or immune-therapy, inhibitors of Galectin-1^96,97^ and -3^98,99^ have been used in combination with anti-CTLA-4 or anti-PD-1 to treat cancer patients in clinical trials.

Because both CD146 and galectins are involved in the modulation of angiogenesis, researchers hypothesized that some galectins may be the ligands of CD146 and the interactions between them are required for functional CD146 in angiogenesis, as well as in cancer metastasis. To date, two galectins, 1 and 3, have been identified as the ligands of CD146.

### Galectin-1

Galectin-1 prefers to bind with the branched *N*-glycans of cell-surface glycoproteins and mediates a glycosylation-dependent angiogenesis.^91,100–103^ It has been reported that increased secretion of Galectin-1 in the ECM facilitates cancer cell proliferation and resistance to cancer therapy in prostate cancer^104^ and Kaposi’s sarcoma.^105^ Mechanistic investigation has revealed that Galectin-1 can bind to *N*-glycans on VEGFR2 to activate VEGF-like signaling in anti-VEGF-A refractory tumors, promoting tumor progression. Accordingly, disruption of the Galectin-1-*N*-glycan axis inhibits tumor growth by promoting vascular remodeling.^101^ This research highlights the importance of Galectin-1 in tumor angiogenesis and cancer metastasis. However, these studies cannot exclude the fact that other cell-surface proteins with branched *N*-glycans are also involved in this glycosylation-dependent pro-angiogenesis pathway.

Early in 2011, it was reported that the co-expression of Galectin-1 and CD146 is required for tumor vascularization in a human mesenchymal stem cell strain with significant angiogenic potential.^106^ In 2013, Jouve et al. reported that Galectin-1 binds to CD146 on endothelial cells, facilitating cell survival.^107^ In this report, they explained that CD146 glycosylation is mainly composed of branched *N*-glycans. They showed that the interaction of CD146 with Galectin-1 is carbohydrate-mediated using both an enzyme-linked immunosorbent assay and surface plasmon resonance assays. In addition, they demonstrated that the interaction between Galectin-1 and CD146 protects endothelial cells against apoptosis induced by Galectin-1. Thus, it is tempting to speculate that CD146 could be a decoy receptor for Galectin-1, preventing the Galectin-1 from binding to pro-apoptotic receptors.^107^ However, whether this interaction affects tumor cell survival remains unknown. In 2015, Yazawa et al. thus further analyzed the functions of this interaction on melanoma and found that when Galectin-1 binds to CD146, it helps maintain intrinsic malignant features.^108^ The authors examined the expression, identity, and function of Galectin-1 ligands in melanoma progression and demonstrated that CD146 is the major Galectin-1 ligand on melanoma cells.

These findings provide a perspective on the interactions between CD146 and its ligands, such as Galectin-1, as contributors to cancer malignancy. Indeed, various membrane glycoconjugates have been identified as binding partners of Galectin-1 such as β1 integrins, CD2, CD3, CD4, CD43, CD45, and GM1 ganglioside. In addition, Galectin-1 can bind to a number of ECM components in a dose-dependent and β-galactoside-dependent manner. For instance, laminin and fibronectin, which are highly *N*-glycosylated, interact with Galectin-1.^109^ Because it has been reported that CD146 can interact with Laminin 411, Laminin 421, and β1 integrin, it is reasonable to speculate that CD146 may also interact with all of those Galectin-1 interactors within cancerous cells.

Since the tumor vasculature is an easily accessible target for cancer therapy, understanding how galectins influence cancer angiogenesis is important for the translational development of therapies intended to prevent tumor progression. Based on the fact that VEGF-targeted therapies often fail when tumors receive continued treatment,^110^ targeting glycosylation-dependent Galectin-1-receptor interactions, such as Galectin-1-CD146-VEGFR2 may increase the efficacy of anti-VEGF treatment.

### Galectin-3

Like Galectin-1, Galectin-3 can also bind to various galactose-terminated glycans of cell-surface receptors and proteins of ECM and is involved in many physiological and pathological processes from cell adhesion and migration to cell activation.^111,112^ In cancer cells, it modulates cell–cell and cell–microenvironment communications, contributing to cancer development, progression, and metastasis.^113–120^ Patients with metastatic diseases tend to have higher concentrations of circulating Galectin-3 than those with only localized tumors.^121^ Increased circulating Galectin-3 promotes blood-borne metastasis due to the interaction of Galectin-3 with receptors on vascular endothelial cells, further causing endothelial secretion of several metastasis-promoting cytokines.

To identify the Galectin-3-binding molecules on the endothelial cell surface, using the Galectin-3 affinity purification method, Colomb et al. found that CD146 was the major cell-surface receptor to strongly bind and co-localize with Galectin-3, compared with other glycosylated receptors, CD31, CD144, and CD106. They also showed that Galectin-3 bound to *N*-linked glycans on CD146 and induced CD146 dimerization and subsequent activation of protein kinase B (AKT) signaling. Correspondingly, suppression of CD146 expression abolishes Galectin-3-induced secretion of metastasis-promoting cytokine from the endothelial cells. Thus, they concluded that CD146 is the functional Galectin-3-binding receptor on the endothelial cell surface responsible for Galectin-3-induced secretion of cytokines, and therefore influences cancer progression and metastasis.^122^

Subsequently, the binding moieties of CD146 by Galectin-3 have been further identified. The authors demonstrated that Galectin-3 interacts with the highly glycosylated Domain 5 in the CD146 extracellular fragment regardless of the presence or absence of lactose. These findings provide a better understanding of how Galectin-3 interacts with cell-surface receptors to mediate endothelial cell migration and the secretion of cytokines.^123,124^

The endothelial galectins are confined to four family members, i.e., Galectin-1, -3, -8, and -9, which contribute to tumor angiogenesis.^92^ Tumor-induced angiogenesis is a pathologic condition in which tumor cells secrete growth factors, such as VEGFs, to promote the growth of new blood vessels.^125,126^ These growth factors activate quiescent endothelial cells in host tissue to facilitate them to invade into the tumor stroma for growth of new capillaries.^127^ Endothelial galectins binding with glycoconjugates on tumors are involved in different processes during tumor-induced angiogenesis. Because Galectin-1 and -3 binding of glycoconjugates on tumor cells mediates many key processes in angiogenesis and elevated levels of Galectin-1 and -3 in the endothelium are correlated with tumor vascularization,^105,128–131^ the promotion of tumor vascular remodeling by tumor CD146 may be due to the interactions between CD146 with Galectin-1 and -3.

### S100A8/A9

#### S100 proteins

In humans, there are at least 21 members of the S100 protein family,^132^ which have 2 EF-hand calcium-binding motifs and are 100% soluble in saturated ammonium sulfate.^133^ S100 family members typically form homodimers, as well as heterodimers, trimers and tetramers, etc.^132,134^ S100 proteins are typically cytoplasmic proteins, but several family members are secreted by cells as extracellular factors.^134–137^ Thus, they contribute to a broad array of intracellular and extracellular functions.^134,135^ Upregulation of S100 proteins promotes pro-inflammatory responses that contribute to the development and progression of cancer and autoimmune and chronic inflammatory diseases.^138–141^

The secreted S100 proteins bind with several cell-surface receptors, including advanced glycation end products (RAGE),^142–146^ toll-like receptor 4 (TLR4),^147^ CD36,^148^ FGFR1,^149^ ALCAM,^150^ CD68,^151^ and ErbB4.^152^ However, how the cell-surface receptors mediate extracellular S100 signaling is lacking and how S100 protein secretion is dynamically regulated in biological processes also still remains unknown.

#### S100A8/A9 heterodimer

The secreted S100A8/A9 proteins are the best characterized soluble S100 proteins. Most inflammatory processes seem to require the release of the S100A8/A9 heterodimer into the ECM.^153–155^ Significant upregulation of S100A8/A9 has been observed in many tumors, including lung, gastric, esophageal, colon, pancreatic, bladder, ovarian, thyroid, breast, and skin cancers.^156,157^ The upregulation of S100A8/A9 is caused either by the infiltrating immune cells of tumor microenvironment^158^ or by the tumor itself,^156,157^ contributing to the establishment of a pre-metastatic niche in the tumor microenvironment.^159^

Mechanistic investigations demonstrated that upregulated S100A8/A9 induces the expression of serum amyloid 3, which in turn recruits myeloid-derived suppressor cell (MDSC), producing a pro-inflammatory environment during metastasis of aggressive disease.^160–167^ In addition, enhanced expression of S100A8/A9 is also associated with poor prognosis.^168^

S100A8/A9 proteins mediate these effects by binding to plasma membrane elements, including heparan sulfate proteoglycan (HSPG),^169^ N-glycans,^170^ TLR4,^171^ and RAGE.^172,173^ In a melanoma lung metastasis model, Hiratsuka et al. clearly demonstrated that lung S100A8/A9, as a strong chemokine, interacts with TLR4 on melanoma to attract distant cancer cells to the lungs.^174^ Recently, it has also been shown that CD146, on melanoma and breast cancer, can respond to lung S100A8/A9 to induce lung-specific metastasis of melanoma^175,176^ and breast cancer.^177^

#### S100A8/A9 as the ligand of CD146

The expression levels of S100A8 and S100A9 were higher in the lungs than in other organs and the higher expression levels were induced by the primary tumor itself.^162^ In lung-associated MDSC and endothelial cells, tumor-derived transforming growth factor-beta (TGF-β) and VEGF-A can upregulate the expression and secretion of S100A8/A9.^162^ Thus, it has been recognized that S100A8/A9 plays a critical role in lung tropic metastasis and the subsequent growth of cancer cells in the lungs.^26,178^ During metastasis, lung S100A8/A9 might act as a guiding protein for cancer cells that possess high expression levels of CD146.^162^

In 2016, Ruma et al. revealed that S100A8/A9 uses CD146 as a receptor during lung-specific metastasis of melanoma cells.^175^ In this study, they demonstrated that S100A8/A9 binding to CD146 activates nuclear factor-kappa B (NF-κB) and induces reactive oxygen species formation, significantly increasing cell adhesion, growth, and invasion. Notably, this study proposed that CD146 governs cancer invasion toward the lungs by sensing the cancer microenvironment as a soil sensor receptor of lung S100A8/A9.^175^ Therefore, the authors conclude that S100A8/A9 plays a crucial role in lung tropic cancer metastasis by helping to establish an immunosuppressive metastatic niche, to which it then attracts remote cancer cells by interacting with CD146 on the cancer cell surfaces.

In 2019, Chen et al. further determined the importance of the S100A8/A9-CD146 axis in melanoma dissemination in a skin lesion, a critical early step for metastasis of melanoma. This mechanistic study revealed that S100A8/A9-CD146 binding activates a cascade of functions; it leads to significant activation of the transcription factor, ETS translocation variant 4 (ETV4), and the subsequent induction of matrix metalloproteinase-25. The activation of MAP3K8/ETV4 by S100A8/A9-CD146 binding finally results in lung tropic metastasis of melanoma.^176^

Breast cancer cells prefer the lung, liver, bone, and brain as their metastatic sites. This organ-tropic metastasis is known as the “seed and soil” theory.^179^ This conclusion was reached because CD146 was remarkably overexpressed in metastatic breast cancer cells.^180–182^ In 2019, in breast cancer cells, the S100A8/A9-CD146 axis-elicited downstream signals that produce the driving force for distant metastasis were identified. This study revealed how S100A8/A9 binding to CD146 accelerates breast cancer growth and metastasis. They found that S100A8/A9 acts as an extracellular cytokine to activate the CD146/ETV4 axis, which upregulates a very high level of ZEB1, a strong EMT inducer. ZEB1 in turn induced a mobile phenotype, i.e., EMT in cells. In contrast, the downregulation of CD146/ETV4 axis repressed S100A8/A9-induced EMT, resulting in greatly weakened tumor growth and lung metastasis. Thus, this report suggested that S100A8/A9 contributes to these signaling processes through CD146.^177^

Since metastasis accounts for the majority of cancer-associated deaths, studies on metastasis mechanisms are needed to establish innovative strategies for cancer treatments. These findings that CD146, as a novel receptor for S100A8/A9, mediates the transition of malignant cancers to metastatic sites, suggest that strategies modulating the interaction between CD146 and S100A8/A9 may be useful for interference with cancer metastasis, especially in the progression of pre-metastatic tumors to the lungs.

### Matriptase

Matriptase is an epithelial-specific membrane-anchored serine protease that proteolytically degrades targets, such as ECM components and the pro-forms of growth factors.^183–186^ Because most of solid tumors are originated from epithelia, matriptase is thus critically involved in cancer invasive growth through degradation events related to breaching the basement membrane, reorganization of the ECM, and activation of oncogenic signaling pathways.^187^

During neurogenesis, matriptase, expressed on neural stem/progenitor (NS/P), plays a critical role in cell-contact signaling between NS/P and brain endothelial cells.^188^ In 2017, the direct binding between brain endothelial CD146 and NS/P matriptase, was identified to be involved in the direct endothelia-NS/P contact.^189^ Such binding can activate the downstream signaling cascades from CD146, including p38 and canonical Wnt/β-catenin pathways in endothelia, leading to secretion of cytokines and chemokines. These factors in turn act on NS/P cells to induce differentiation and migration for the adequate neurogenesis. Consistently, none of these signaling events occurred when either matriptase or CD146 is deleted. Thus, this study suggests that CD146, expressed on stromal cells in tumor microenvironment, plays essential roles in tumor invasion by interaction with matriptase expressed on invading cancer cells originated from epithelia.

Recent years, matriptase has received considerable attention in the field of cancer research, because it is upregulated in many cancers and is required for the degradation of the ECM and the maturation of a variety of oncogenic pro-growth factors.^184^ Mice with reduced levels of matriptase display a significant delay in oncogene-induced mammary tumor formation and growth.^190,191^ Therefore, interference of the interaction between CD146 on stromal cells and matriptase on cancers is a reasonable strategy to turn off the signaling pathway and invasive responses in cancers, especially epithelia-originated neoplasms.

## CD146 is the co-receptor of pro-angiogenic factor receptors

**Fig. 2:**
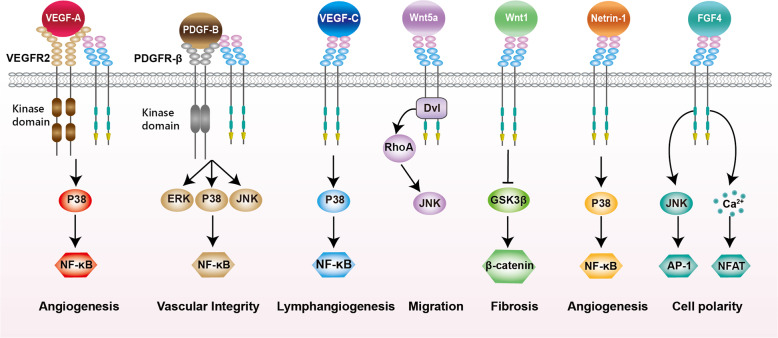
Schematic representation of CD146 as the co-receptors of growth factor receptors or growth factors. The detailed functions elicited by the interactions between CD146 and these ligands are described in text

Angiogenesis refers to the physiological process by which new blood vessels are formed from preexisting blood vessels. This highly ordered process relies on extensive signaling networks both among and within endothelial cells, their associated mural cells (vascular smooth muscle cells and pericytes) and other cell types (e.g., immune cells). VEGF-A is the principal mediator of angiogenesis and contributes to the formation of a pioneering tip cells of angiogenic sprouts.^192^ With further vessel maturation, endothelial cells-secreted platelet-derived growth factor (PDGF)-B is the major player for recruitment of adjacent pericytes and vascular smooth muscle cells to the endothelial surface.^193,194^ We found that CD146 can directly interact with VEGFR2^53^ and PDGFR-β^195^ to promote tumor angiogenesis and cerebrovascular development, respectively (Fig. 2).

### CD146 as the co-receptor for VEGFR2 in tumor angiogenesis

There are five members of the human VEGF family: VEGF-A, -B, -C, -D and placental growth factor (PlGF).^196,197^ VEGF-A is the best characterized family member and the most potent stimulator of angiogenic processes.^192^ VEGF-A has at least nine different splicing forms with different binding affinities within its receptors and ECM components.^198^ VEGF-A is secreted by many cell types, such as endothelial cells,^199,200^ fibroblasts,^201^ smooth muscle cells,^202^ platelets,^203^ neutrophils,^204^ macrophages, and ~60% of all tumors.^205^ VEGF-A secretion is induced by ischemia and inflammatory stimuli.^206^ Cellular responses to VEGF-A are mainly driven by their binding to its cognate receptors—the VEGFRs.

VEGFRs belong to the class IV receptor tyrosine kinase family and are expressed by endothelial cells, macrophages, hematopoietic cells and smooth muscle cells.^207–210^ In humans, there are three VEGFR subtypes, which are encoded by separate genes: *VEGFR1 (FLT1)*, *VEGFR2 (KDR)*, and *VEGFR3 (FLT4)*. Among them, VEGFR1 and VEGFR2 show high structural similarity.^198,211^ In endothelial cells, VEGFR2, but not VEGFR1, mediates the full range of VEGF responses.^212,213^

VEGFR2 activation leads to distinct activation patterns, including proliferation via mitogen activated protein kinase (MAPK),^214^ cell migration via phosphoinositide-specific phospholipase C (PLC)-γ^215^ and focal adhesion kinase (FAK),^216,217^ and cell survival through phosphatidylinositol-4,5-bisphosphate 3-kinase (PI3K)/AKT.^218^ In addition, VEGFR2 interaction with its co-receptors is essential for its functions. Till now, the well-established VEGFR2 co-receptors include Neuropilin-1, CD44, vascular endothelial cadherin, and β integrins.^219^ Although the exact molecular mechanisms of how VEGFR2 induces diverging downstream signals have not been completely understood, ligand diversity and availability as well as interactions with co-receptors might explain most of these effects.

Similar to the functions of VEGFR2 in endothelia, CD146 also contributes to endothelial cell proliferation, migration, and angiogenesis.^220–222^ Different from VEGFR2 with kinase activity, CD146 does not show any kinase activity. But similarly to VEGFR2, CD146 also can activate numerous signaling pathways, known as the downstream cascades of VEGFR2, such as focal adhesion formation mediated by FAK,^14^ cell migration mediated by PLC-γ,^14^ cell survival through PI3K/AKT,^223^ proliferation via MAPK and NF-κB.^224^

In 2012, we found that CD146 interacts with VEGFR2 on endothelia and acts as a co-receptor.^53^ This interaction occurs in the extracellular protein domain of CD146 independently of VEGF-A. When CD146 was inhibited using the blocking antibody AA98 or CD146 siRNA, VEGF-A induced phosphorylation of VEGFR2 was suppressed in human umbilical vein endothelial cells (HUVECs), demonstrating that the interplay of CD146 with VEGFR2 is mandatory for functional VEGFR2 signaling.

In addition to the extracellular binding of CD146 to VEGFR2, intracellular CD146 signaling is also required for VEGF-A-induced signal transduction via VEGFR2. When associated with VEGFR2, the cytoplasmic tail of CD146 recruited ERM proteins and the actin cytoskeleton, to assemble a “signalosome,” which is required for signal transduction from VEGFR2 to AKT and P38 MAPK. Inhibition of CD146 by blocking antibody AA98 hinders the interaction between VEGFR2 and CD146, resulting in abrogation of the downstream cascade of p38 MAPK and AKT signaling. Therefore, our study revealed that the interaction of CD146 with VEGFR2 improves pro-angiogenesis signaling as part of such a signalosome in which CD146 binding to VEGFR2 enables the reorganization of the cytoskeletal network during angiogenesis.

Using a preclinical pancreas carcinoma xenograft model, we found that the efficacy of combined treatment with the anti-VEGF antibody Bevacizumab and the anti-CD146 antibody AA98 was significantly higher than treatment with either one of the two agents. The clinical benefit from VEGF-targeted therapies is always compromised during continued treatment with anti-VEGF antibodies, such as Bevacizumab in pancreatic carcinoma.^110,225,226^ Thus, our findings indicate the possibility that the clinical efficacy of anti-VEGF therapy can be augmented by targeting CD146 treatment in the future.

Several mechanisms by which tumor angiogenesis may proceed in the presence of anti-VEGF have been identified. For example, pro-inflammatory cytokines and Galectin-1 produced from tumors activate VEGF-like pro-angiogenic pathways.^101,227^ Based on our findings, we speculate that the interaction between CD146 and VEGFR2 might also underlie the mechanisms of anti-VEGF treatment failure. Thus, in the future, investigating the interaction network of Galectin-1, CD146, and VEGFR2 will be necessary for a better understanding of the mechanisms underlying the angiogenesis and metastasis of carcinoma.

### CD146 as the co-receptor of PDGFR-β in vessel integrity

PDGF family members, including PDGF-A, -B, -C, and -D, play a crucial role in embryologic development based on the fact that all PDGF knockout mice are embryonic lethal. Similar to VEGFs, all PDGFs are dimers of disulfide-linked polypeptide chains.^228^ In the mouse embryo, PDGF-B is strongly expressed in the developing vascular endothelium,^229^ tip cells of angiogenic sprouts, and in the endothelia of growing arteries, where pericytes are actively recruited.^230,231^ During angiogenesis, PDGF-BB released from endothelial cells promotes the recruitment of adjacent pericytes to the endothelial surface.^193,194^

Both PDGFs and their receptors (PDGFR-α and -β) are implicated in tumor angiogenesis and lymphangiogenesis.^232–235^ PDGF-AA and PDGF-CC mainly bind to PDGFR-α, whereas PDGF-BB binds to PDGFR-β.^236^ PDGFR-β is expressed in the mesenchyme, particularly in vascular smooth muscle cells and pericytes.^230,237^ PDGF-BB/PDGFR-β signaling elicits several well-characterized signaling cascades, such as Ras-MAPK, PI3K, and PLC-γ. The PDGFR-β-Ras-MAPK cascade leads to the stimulation of cell proliferation, differentiation, and migration.^238,239^ The PDGFR-β-PI3K branch promotes cell growth and cytoskeletal reorganizations.^240^ PDGFR-β-PLC-γ activates intracellular Ca^2+^ mobilization, stimulating Ca^2+^-dependent secondary signaling, such as tube formation and cell motility.^241,242^ In addition, in adult mice, PDGF-BB/PDGFR-β signaling exerts a neuroprotective function by the recruitment of pericytes.^243^

In 2017, we revealed that CD146 controls pericyte recruitment and vessel stabilization through interactions with PDGFR-β to protect the CNS.^59^ First, we extensively examined the expression patterns of CD146 in brain endothelial cells and pericytes throughout development and adulthood. In mouse brains, CD146 is first expressed in the cerebrovascular endothelial cells of premature blood vessels without pericyte coverage; with increased coverage of pericytes, CD146 could not be detected in cerebrovascular endothelial cells, it could only be found in pericytes. Furthermore, we studied the role of CD146 in endothelial-pericyte communication through its selective deletion in both cell types. We found that a knockdown in either cell type leads to the breakdown of the blood-brain barrier (BBB), which initiates the invasion of the endothelial cells toward the CNS and the recruitment of pericytes to the nascent vessels during embryogenesis. Furthermore, we demonstrated that CD146 functions as a co-receptor of PDGFR-β to mediate pericyte recruitment to cerebrovascular endothelial cells, indicating that pericyte CD146 was important for pericyte recruitment and vessel stabilization. The attached pericytes in turn downregulate endothelial CD146 by secreting TGF-β1 to promote further BBB maturation.

Because both CD146 and PDGFR-β are involved in regulation of growth and survival of different cell types, including cancer cells, further investigation of functions of CD146 and PDGFR-β interaction in cancers may help deeply understand the dysregulation of spatio-temporally controlled PDGF-BB induced signaling during tumor development and progression, especially tumor angiogenesis and lymphangiogenesis.

## CD146 is the receptor for growth factors

Interestingly, all the ligands identified by our laboratory are well-known growth factors and mitogens, which include Wnt5a, Wnt1, Netrin-1, FGF4, and VEGF-C. These ligands and their cognate receptors are not only involved in almost all cellular processes required for embryogenesis, development, and adult life, but also effective targets for numerous anticancer treatments (Fig. 2).

### CD146 as the receptor of VEGF-C in lymphangiogenesis

VEGF-C belongs to the VEGF family.^197,244^ Different from VEGF-A in angiogenesis, VEGF-C is the principal driver of lymphangiogenesis and controls the whole process of lymphangiogenesis.^245–247^ Genetic studies in mice have revealed that loss of VEGF-C impairs the development of the lymphatic vasculature.^247,248^ On the other hand, overexpression of VEGF-C in specific tissues induces in situ lymphangiogenesis, such as in the skin,^249,250^ pancreas,^251^ and lung.^252^

Full-length VEGF-C undergoes proteolytic processing and becomes a fully processed form, which increases its affinity for VEGFR2 and VEGFR3.^253,254^ VEGF-C regulation of proliferation and migration of lymphatic endothelial cells is mainly through binding with VEGFR3.^255–257^ VEGF-C/VEGFR3 activates the ERK-MAPK and PI3K/AKT pathways and enhances diverse physiological effects in lymphatic endothelial cells, such as growth, proliferation, mobility, and invasiveness. Therefore, the signaling axis of VEGF-C/VEGFR3 is under extremely sophisticated control.^258–260^ However, the receptors that mediate VEGF-C signal transduction for lymphatic sprouting, the initiating step of lymphangiogenesis, remain elusive.

In 2017, we found that VEGF-C is also the ligand of CD146 and the binding site exists between D4 and D5 domains of the extracellular domain of CD146.^57^ VEGF-C activates CD146 to mediate sprouting during lymphangiogenesis, although the exact amino acid residues in the intracellular domains of CD146 responsible for transmitting VEGF-C signals still need to be defined. Using a zebrafish model, we discovered why CD146 is required for the lymphatic sprouting during development. Knockdown of CD146 inhibited phosphorylation of p38 and ERK, while knockdown of VEGFR3 inhibited phosphorylation of AKT and ERK. Furthermore, we confirmed that inhibition of p38 mainly reduced sprouting of lymphatic endothelial cells during lymphangiogenesis.

Maladjustment of VEGF-C-elicited signaling results in tumor metastasis, especially via lymphatic vessels.^261–265^ Lymphatic metastasis is a challenge for clinical treatment of tumors and is the cause of death for some cancers,^266,267^ such as breast cancer,^268^ lung cancer,^269^ and melanoma.^270^ Soluble VEGFR3,^271,272^ VEGF-C inhibitor,^273^ VEGFR3 antibodies,^274^ and VEGF-C siRNA^275^ have been used in the treatment of lymphatic metastasis. Our findings suggest that targeting CD146 may also be an effective therapeutic strategy to treat lymphatic metastasis.

### CD146 as the receptor of Wnt5a in cell migration

The Wnt family includes several secreted glycoproteins that are involved in the regulation of a wide variety of normal and pathological processes, including embryogenesis, differentiation, and tumorigenesis.^276^ In human, there are 19 *wnt* genes that encode functionally distinct Wnt proteins, which can bind to their receptors, FZD/LRP heterodimers. Wnt signaling pathways include canonical and non-canonical cascades. The canonical pathway causes stabilization and nuclear translocation of β-catenin, which regulates transcription of Wnt target genes. The non-canonical pathway is β-catenin-independent and can be further divided into Wnt/planar cell polarity (PCP), Wnt/Ror2, and Wnt/Ca^2+^ signaling cascades.^277^ The PCP pathway is activated by c-Jun N-terminal kinases (JNKs).^278^

Wnt5a transmits signals through either canonical or non-canonical Wnt pathway.^279,280^ In 2013, we found that CD146 is the receptor of Wnt5a and is required for the Wnt5a-controlled cell migration and convergent extension during zebrafish embryogenesis.^54^ The biochemical experiments revealed that CD146 binds to Wnt5a with the high affinity, leading to activation of JNK-PCP pathway and downregulation of β-catenin expression.^54^ Further analysis demonstrated that CD146 can interact with Dvl2, and this interaction is enhanced under Wnt5a treatment. Accordingly, knockout of CD146 results in dysregulation of the Wnt/PCP pathway. Thus, our findings provide the first direct evidence that CD146 turns on the non-canonical Wnt signaling branch as a functional Wnt5a receptor in cell migration during development.

Wnt5a is upregulated in various types of human cancers.^281,282^ Meanwhile, Wnt5a activation of JNK is linked with cytoskeletal remodeling and cell motility in various cell systems.^283–285^ For example, in melanoma, Wnt5a is thought to directly affect cell motility and metastasis.^286^ In this view, CD146 may represent the prime target to develop more effective and less toxic therapies toward Wnt5a/CD146/JNK activation for meeting the challenges from tumor metastasis.

### CD146 as the receptor of Wnt1 in fibroblast activation

*Wnt1*, originally known as oncogene *int-1*, was initially discovered by analysis of host cell sequences adjacent to viral integration sites in tumors of mice infected with mouse mammary tumor virus.^287,288^ Subsequent evidence suggests that the oncogenic functions of Wnt1 is mediated via upregulation of proliferative genes by canonical β-catenin pathway.

Similar with CD146, Wnt1 protein expression levels are high at developmental stage and low in adults, and ectopic expression of Wnt1 causes tumor development.^289^ In 2018, we found that CD146 can directly bind with Wnt1 in fibroblast, activating fibroblast via canonical Wnt/β-catenin pathway. Such interaction is essential for Wnt1-induced fibroblast proliferation and ECM production.^58^

Because cancer-associated fibroblasts (CAFs), as the main cellular constituent of the cancer-associated stroma, can drive cancer cell invasion but can also impair the migration and activation of T cells. Herein, researchers should further examine whether CD146 is a marker of CAFs, and if so, inhibiting the interaction of CD146 with Wnt1 on CAFs may benefit cancer treatment.

### CD146 as the receptor of Netrin-1 in angiogenesis

Netrin-1 belongs to a family of Laminin related secreted proteins that control axonal and cellular migration during the development of the nervous system.^290–293^ Netrin-1 is a 640 amino acid protein and its carboxy-terminal sequence is enriched in basic amino acids.^294–296^ This sequence can bind HSPGs with high affinity, contributing to retaining them in the ECM.^297^ Netrin-1 is not only expressed in the nervous system but also in other non-neuronal organs.^290,291,298^ It regulates diverse processes, such as neuronal navigation, cellular adhesion, motility, proliferation, and differentiation during development.^299–302^ Dysregulation of Netrin-1 is involved in diverse pathological processes, such as cancer, cardiovascular disease, and kidney disease, making it an attractive potential therapeutic target.^301,303,304^

Netrin-1 acts through two main receptors, DCC (deleted in colorectal cancer) and UNC5B (uncoordinated-5 homolog), to alter the architecture of the cytoskeleton networks.^305,306^ Binding to its receptors activates chemotropic responses and adhesive mechanisms, regulating inflammation, angiogenesis, and apoptosis.^299,301,302^

Our laboratory clarified that netrin-1 binds CD146 with a higher affinity than the classical UNC5B receptor.^55^ Netrin-1 preferentially binds CD146 at low concentrations (50–200 ng/mL) and binds UNC5B at high concentrations (1000–2000 ng/mL). In addition, our study demonstrated the dual action of Netrin-1 on angiogenesis: the pro-angiogenic roles through CD146 and the antiangiogenic functions through UNC5B. CD146 silencing or deletion inhibits Netrin-1-induced HUVEC proliferation, migration, and tube formation, as well as VEGFR2, p38, and extracellular regulated MAP kinase (ERK)1/2 activation. In contrast, Netrin-1 binding with UNC5B at high concentrations triggers signals that counteract the CD146-mediated pro-angiogenic pathway. CD146 can also interact with VEGFR2 as a co-receptor;^53^ because of this, CD146-Netrin-1 binding may further improve VEGF-A signaling-mediated angiogenesis.

Thus, this finding highlights the importance of CD146 in Netrin-1-induced angiogenesis, although other factors are also likely contributors in the absence of CD146.^303^ Similarly to CD146, Netrin-1 also takes part in tumor growth, these properties of Netrin-1 in cancer offer reasonable therapeutic approaches. Therefore, preclinical and clinical studies should be planned to investigate the therapeutic potential of the Netrin-1/CD146 pathway in tumor angiogenesis.

### CD146 as the receptor of FGF4 in apical-basal polarization

FGFs were initially recognized as fibroblast-specific growth factors, and it is now recognized that FGFs regulate multiple biological functions in diverse cell types beyond fibroblasts. FGFs constitute a large family with 23 members,^307^ which share sequences and structural similarities.^308^ These factors include paracrine or endocrine forms and participate in important pathophysiological processes such as cell proliferation, survival, migration, angiogenesis, wound healing, differentiation, and endocrine secretion regulation during development and adult life.^309–311^

Among FGFs, only FGF4 and eight have been revealed to possess the chemotactic activity and FGF4 acts as a chemo-attractant during morphogenesis.^312^ FGF4 belongs to the paracrine FGFs that signal FGFRs by forming a tripartite complex with FGFRs (1–4) and HSPGs.^313^ Although extensive investigations focus on the FGF signaling, there has been no indication that FGF ligands can bind with other receptors beyond FGFRs. Meanwhile, no direct evidence has indicated that any one of the FGFRs mediates the FGF4-elicited chemo-attracting activities.

The chief intracellular substrates of FGF signaling are FGF receptor substrate 2 (FRS2) and PLC-γ. Activated FRS2 activates the transcription factors of activator protein 1 (AP-1) and forkhead box protein O (FOXO) through RAS–ERK or the PI3K-pyruvate dehydrogenase kinase pathway, respectively, whereas PLCγ leads to the activation of Ca^2+^-dependent nuclear factor of activated T cells (NFAT).^313^ There are several points where other pathways can cross-talk with FGF signaling, for instance, activated JNK is required for AP-1 activation and FOXO nuclear translocation.^314^

In 2017, we found that CD146 is an independent receptor of FGF4.^56^ The binding affinity between CD146 and FGF4 is higher than that between FGF4 with its receptors (FGFR1-4). In addition, the presence of HSPG is not required for the binding of FGF4 to CD146. Using zebrafish as the model system, we found that CD146, but not FGFR, is the responsive receptor for FGF4. It provided local spatial cues to organize apical-basal polarity in participating cells during morphogenesis. An in vitro lumen formation assay further confirmed the migration of CD146, but not FGFR1, toward FGF4 as the key activity during FGF4-induced establishment of the apical-basal polarity. By investigating the effects of CD146 on FGF signaling output, we found that the cooperative actions between CD146-dependent activation of JNK and NFAT together with FGF signaling-dependent activation of ERK ensure that CD146^+^ cells concomitantly upregulate the transcriptional activity of AP-1 and NFAT during organ morphogenesis.

Thus, our findings suggest the essential roles of CD146 in FGF4-elicited morphogenetic signaling. In light of the coincident spatiotemporal distribution of CD146/FGF4 on developmental embryos, CD146 may be a more preferable receptor in FGF4 biological responses than FGFRs. The cooperation of CD146*/*FGF4 in cell polarity establishment suggests that CD146 could be the genuine responsive receptor in all of FGF4-executed chemo-attracting actions. Because FGF4 possesses the potent pro-angiogenetic activity and fails to bind heparan sulfate in the heart and blood vessels,^315^ it is possible that CD146-FGF4 is also a critical partner in angiogenesis. For this reason, in the therapeutic regimes of pathological angiogenesis, simultaneous targeting of both CD146 and FGF4 could have better efficacy than settings with that singular target either.

## Conclusions

Overall, CD146 expression is frequently increased in fast-proliferating cells, such as cells in their developmental stages and cancer cells.^21^ From the functional perspective of CD146 as a CAM, upregulation of CD146 expression enhances the interactions between CD146 and its ligands in the ECM, including Laminin 411, Laminin 421, Galectin-1, Galectin-3, S100A8/A9, and matriptase, shifting the balance between cell–cell and cell-matrix adhesion while increased secretion of pro-metastatic cytokines causes cells to invade their surrounding ECM, spread via the vascular or lymphatic circulation, and extravasate into distant organs. As a co-receptor of VEGFR2 and PDGFR-β, or an independent receptor of growth factors of Wnt5a, Wnt1, Netrin-1, FGF4, and VEGF-C, highly expressed CD146 can enhance pro-angiogenesis signaling and promotes cell growth, proliferation, differentiation, and survival. Therefore, these novel scenarios highlight the importance of CD146 in proliferating cells and facilitate a better understanding of the mechanisms and implications of the interactions between CD146 with its ligands in invasive growth, proliferation, and motility of cancer cells.

It has been reported that there are three blocking antibodies to membrane CD146; ABX-MA1 raised by Bar-Eli’s laboratory, AA98 raised by our laboratory, and TsCD146 raised by Blot-Chabaud’s laboratory. They exhibit powerful inhibitory activity on membrane CD146’s function. ABX-MA1 can effectively decrease cell–cell adhesion and cell invasion in vitro, as well as decrease primary tumor growth and lung metastases in vivo.^316–318^ AA98 displays prevailing inhibitory activity on cancer progression in various models of tumor bearing mice.^181,319–322^ TsCD146 can specifically recognize and internalize cancer CD146 without interfering with physiological CD146 on vessels, suggesting great potential in tumor diagnostic and/or therapeutic applications.^28^ Because of this, CD146-targeted immunotherapy has promising therapeutic value in tumor treatment because manifold dose regimens of the antibodies could be administered to the patients without increasing the immune reaction.

Immunotherapy, including immune checkpoint blockades, has revolutionized the field of cancer therapy in the last decades. However, due to the inability of T cells to access the tumor microenvironment, there are still a substantial number of patients do not benefit from current forms of immunotherapy. Given the various roles of CD146 in the remodeling tumor microenvironment, immunotherapy against CD146 could provide a possibility for overcoming this impediment. Metastasis accounts for the majority of cancer-associated deaths, therefore establishing innovative strategies for modulating CD146 and ligand interactions are needed for cancer treatment in the future.
